# Oculomotor atypicalities in Developmental Coordination Disorder

**DOI:** 10.1111/desc.12501

**Published:** 2016-10-17

**Authors:** Emma Sumner, Samuel B. Hutton, Gustav Kuhn, Elisabeth L. Hill

**Affiliations:** ^1^ Department of Psychology, Goldsmiths University of London UK; ^2^ School of Psychology University of Sussex UK

## Abstract

Children with Developmental Coordination Disorder (DCD) fail to acquire adequate motor skill, yet surprisingly little is known about the oculomotor system in DCD. Successful completion of motor tasks is supported by accurate visual feedback. The purpose of this study was to determine whether any oculomotor differences can distinguish between children with and without a motor impairment. Using eye tracking technology, visual fixation, smooth pursuit, and pro‐ and anti‐saccade performance were assessed in 77 children that formed three groups: children with DCD (aged 7–10), chronologically age (CA) matched peers, and a motor‐match (MM) group (aged 4–7). Pursuit gain and response preparation in the pro‐ and anti‐saccade tasks were comparable across groups. Compared to age controls, children with DCD had deficits in maintaining engagement in the fixation and pursuit tasks, and made more anti‐saccade errors. The two typically developing groups performed similarly, except on the fast speed smooth pursuit and antisaccade tasks, where the CA group outperformed the younger MM group. The findings suggest that children with DCD have problems with saccadic inhibition and maintaining attention on a visual target. Developmental patterns were evident in the typically developing groups, suggesting that the pursuit system and cognitive control develop with age. This study adds to the literature by being the first to systematically identify specific oculomotor differences between children with and without a motor impairment. Further examination of oculomotor control may help to identify underlying processes contributing to DCD. A video abstract of this article can be viewed at: https://youtu.be/NinXa2KlB4M. [Correction added on 27 January 2017, after first online publication: The video abstract link was added.]

## Research highlights

- This study is the first to use eye tracking technology to assess both fundamental eye movements and higher order processes involved in oculomotor control in children with DCD.
- Low level oculomotor processes appear intact in children with DCD, alongside typically developing groups.
- Difficulties with exerting top‐down cognitive control were evident in children with DCD and a younger typically developing group.
- Developmental effects were found for the two typically developing groups, with older children performing better than younger children on duration of smooth pursuit and antisaccade performance which measures inhibition.

## Introduction

Developmental Coordination Disorder (DCD) is a neurodevelopmental disorder characterized by significant difficulties with the acquisition and execution of motor skill (DSM‐5, American Psychiatric Association [APA], 2013); and has an estimated prevalence between 2 and 5% (Kirby & Sugden, 2007; Lingham, Hunt, Golding, Jongmans & Edmond, 2009). Individuals with DCD demonstrate a level of motor skill out of keeping with their age and intellectual ability. Moreover, problems with acquiring adequate motor skill significantly interfere with activities of daily living (e.g. dressing, eating) and educational attainment and, importantly, cannot be accounted for by a sensory or neurological impairment, or general medical condition.

In most cases, motor tasks are visually guided. Successful completion of such tasks is dependent on interactions between vision and proprioception and importantly requires accurate, up‐to‐date visual feedback to guide limb movements. Although high in acuity, the foveal region is very small (Tresilian, 2012); therefore, the oculomotor system must programme eye movements to move the fovea to the relevant location of interest. Fundamental oculomotor behaviour includes fixations, saccades and smooth pursuit. Fixation orients a stationary target, whilst smooth pursuit is a directed tracking movement to maintain a moving target on the centre of the fovea (Karatekin, 2007). Saccades, a rapid eye movement, also aim to centre our fovea on the object of interest as we navigate through the environment. These fundamental oculomotor processes allow accurate visual feedback to enable successful motor activity.

Two studies have specifically examined oculomotor function in DCD, both focusing on the pursuit system. Smooth pursuit develops very early in infancy (von Hofsten & Rosander, 1997). When the pursuit system is immature, lower gain (i.e. the ratio of eye velocity to target velocity) will be evident and catch‐up (compensatory) saccades are employed for the eye to recapture the target. With maturation, compensatory saccades become less frequent (Ingster‐Moati, Vaivre‐Douret, Quoc, Albuisson, Dufier *et al*., 2009). An early study that recorded eye movements using infrared limbus reflection technique reported that children with a diagnosis of DCD (*n* = 8) had significantly lower horizontal pursuit gain than a typically developing control group (Langaas, Mon‐Williams, Wann, Pascal & Thompson, 1997). Greater unsigned lag (i.e. less synchronization with the stimulus) was also found in the DCD group, as these children made more high velocity shifts (saccades) away from and toward the target; leading the authors to conclude that children with DCD present with a poorly defined predictive model of the target motion. This finding lends some support to the ‘internal modelling deficit’ hypotheses of DCD (see Adams, Lust, Wilson & Steenbergen, 2014), which proposes a difficulty with generating or using predictive estimates of motor control (i.e. forward modelling). However, in contrast, using electrooculography (EOG), Robert, Ingster‐Moati, Albulsson, Cabrol, Golse *et al*. (2014) found that children with DCD were comparable in pursuit gain to a group of children without a DCD diagnosis on a horizontal pursuit task, whereas vertical pursuit gain was poorer in those with DCD. Further investigation of the oculomotor system may help to clarify whether difficulties with internal modelling are truly characteristic of DCD.

Disparity between the two studies could be due to developmental differences, as children were aged 5–7 years in Langaas *et al*. (1997) and 7–12 years in Robert *et al*. (2014). Also, a third of the DCD sample in Robert *et al*.'s study had undergone intervention by orthoptists; although analyses of these children separately from those without intervention showed only an advantage in the vertical pursuit task. Langaas *et al*. provide the more detailed account, revealing that young children with DCD make both anticipatory and corrective saccades when tracking a target, which suggests that these children are not always in pursuit of the target. However, both of these studies are limited in that motor competency was not specifically tested, and thus not confirmed in the control group. Moreover, attention difficulties, which are often reported in DCD (Dewey, Kaplan, Crawford & Wilson, 2002), were not assessed or controlled for. Eye tracking studies of children with attention‐deficit‐hyperactivity disorder (ADHD) also highlight oculomotor deficits in comparison to those without a diagnosis of ADHD (Hanisch, Radach, Holtkamp, Herpertz‐Dahlmann & Konrad, 2006; Mahone, Mostofsky, Lasker, Zee & Denckla, 2009). It is possible that the presence of ADHD‐like symptoms influenced the previous results in some way.

The saccadic system has been studied extensively in neuropsychiatric disorders such as schizophrenia (Hutton, Huddy, Barnes, Robbins, Crawford *et al*., 2004), and other neurodevelopmental disorders such as ADHD (Hanisch *et al*., 2006; Mahone *et al*., 2009) and autism spectrum disorders (ASD; Kelly, Walker & Norbury, 2013; Mosconi, Kay, D'Cruz, Seidenfeld, Guter *et al*., 2009; Takarae, Minshew, Luna & Sweeney, 2004). A range of tasks can be used to assess saccades. The *prosaccade task* measures an individual's ability to generate a saccade towards peripheral targets and is indicative of sensorimotor function; while the more demanding *antisaccade task* relies on top‐down cognitive processes, requiring the individual to suppress a prosaccade towards the peripheral target and instead shift their gaze in the opposite direction. Inhibition is a crucial developmental executive function and inefficiency in inhibitory control may have negative repercussions on skill acquisition (Johnson & de Haan, 2015). Recent reviews of executive functions in children with DCD have revealed inhibition difficulties, although varying methodologies and task demands (verbal/motor/visuospatial) make comparisons difficult (see Leonard & Hill, 2015; Wilson, Ruddock, Smits‐Engelsman, Polatajko & Blank, 2013). A study examining inhibition using separate verbal and motor tasks demonstrated that children with DCD produced more errors in a motor inhibition task but were slower at inhibiting a verbal response (Bernardi, Leonard, Hill & Henry, 2015). These findings implicate the cognitive control system in DCD and could usefully be extended with an assessment of antisaccade eye movements which have the added benefit of eliminating potential confounds (i.e. verbal and motor demands).

Surprisingly little is known about the integrity of the oculomotor system in individuals with DCD. Given that a wealth of studies have looked into neural correlates of the oculomotor system (Karatekin, 2007), investigation into this area may provide insight into the underlying neural characteristics of DCD. Thus the present study was designed to provide a detailed account of oculomotor performance in children with DCD. A comprehensive battery was employed to measure: (a) fixation stability, (b) horizontal smooth pursuit (at two speeds: slow and fast), and performance on (c) pro‐ and (d) anti‐saccade tasks. This generates a full profile of the lower level fundamental processes involved in oculomotor control (fixations, saccades, smooth pursuit) as well as considering higher order control (antisaccades).

To better understand the specificity of any oculomotor anomalies within DCD, two typically developing comparison groups were recruited; the first matched to children with DCD (7–10 years) by chronological age (hereafter ‘CA’ group), and the second matched to the DCD group by motor ability (motor‐match, hereafter ‘MM’ group). Inevitably, the MM group was younger than children with DCD. Inclusion of the MM group extends the current literature and can help to determine whether children with DCD show immaturity or, in fact, atypicalities in the oculomotor system. Although children with additional diagnoses (such as ADHD) were systematically excluded from the study, hyperactivity was assessed as it has been reported that children with DCD have elevated symptoms (Kirby & Sugden, 2007; Saban, Ornoy & Parush, 2014) and these might in themselves influence oculomotor findings (see Karatekin, 2007).

## Method

### Participants

Participants formed three groups: children with DCD, typically developing CA matched children, and the typically developing MM group. The following selection criteria/background measures applied to all groups (see Table 1 for the results of points 1–3):

1. All participants had a measured Full Scale IQ (FSIQ) ≥80 (DCD & CA: Wechsler Intelligence Scale for Children [WISC‐IV; Wechsler, 2003]; MM: Wechsler Preschool and Primary Scale of Intelligence [WPPSI‐IV; Wechsler, 2012]).
2. Motor competency was assessed using the Movement Assessment Battery for Children (MABC‐2; Henderson, Sugden & Barnett, 2007). Overall test performance was converted to a percentile rank (UK norms). As all children in the DCD group had an existing diagnosis, the MABC‐2 inclusion criterion for this group was a score ≤16th percentile. Children in the CA and MM groups scored ≥25th percentile.
3. Although no child had a diagnosis of ADHD, class teachers completed the Strengths and Difficulties Questionnaire (SDQ; Goodman, 1997) for each child, which includes five questions about hyperactivity/concentration. No cut‐off was used, but children with DCD scored significantly higher on this scale than the control groups (*p* < .001, see Table 1).
4. When completing a background screening questionnaire, parents of children with DCD reported no additional diagnoses, such as ADHD, ASD, or dyslexia, no form of visual or neurological impairment, or general medical condition; and parents of children in the CA and MM groups did not identify diagnoses of any kind.

**Table 1:** Background characteristics of the three groups

	CA (*n* = 25)	DCD (*n* = 23)	MM (*n* = 29)	*F*	*p*	Post hoc
Gender (m;f)	19;6	15;8	19;10	–	–	–
Age (in years)						
Mean (*SD*)	9.00 (.96)	8.94 (1.20)	6.18 (.65)	70.36	< .001	(CA=DCD) > MM
Range	7–10	7–10	4–7			
FSIQ standard score						
Mean (*SD*)	109.04 (10.69)	101.52 (11.56)	109.34 (11.82)	3.67	.05	CA = DCD = MM
Range	89–124	87–126	88–140			
MABC2%ile						
Mean (*SD*)	65.40 (21.55)	3.51 (5.22)	45.21 (19.73)	15.02†	< .001	DCD < MM < CA
Range	25–98	.01–16	25–91			
SDQ hyperactivity						
Mean (*SD*)	1.88 (1.86)	6.36 (2.59)^a^	2.14 (2.25)	29.32†	< .001	(CA=MM) < DCD
Range	0–6	0–10	0–7			
Peg placing (secs)						
Mean (*SD*)	30.13 (4.96)	41.39 (8.06)	40.10 (4.38)	29.01	< .001	(DCD=MM) > CA
Range	22–39	31–61	33–61			

*Note*:

FSIQ = Full Scale IQ from WISC/WPPSI, *M* = 100, *SD* = 15. MABC2 = Movement Assessment Battery for Children, *M* = 10, *SD* = 3; SDQ = Strengths and Difficulties Questionnaire; SDQ hyperactivity scoring: 0–5 = ‘average’, 6–7 = ‘slightly raised’, 8 = ‘high’, 9–10 = ‘very high’. ^†^Nonparametric analyses conducted due to unequal variances (Kruskal‐Wallis *H* and post‐hoc Mann‐Whitney reported). ^a^2 substituted data points (2 parent SDQ responses were used instead of teacher responses because they failed to return the questionnaire).

Thirty‐four children with DCD, aged 7‐10 years, were initially recruited through primary schools in South London and by advertisements through a charity, the Dyspraxia Foundation. As a result, children from many locations in England (North, Midlands, and South of England) took part in the study, reflecting a range of socioeconomic backgrounds and strengthening the study in terms of generalizability. Children had an existing diagnosis of DCD from a multi‐disciplinary team of clinicians based in their home county and external to the research team. One child was excluded due to an FSIQ score below cut‐off, four more children were excluded because they scored above cut‐off on the motor assessment, and six children had difficulty remaining still and completing the calibration procedure on the eye tracker and thus did not proceed to complete the full assessment. Therefore, the final sample comprised 23 children (15 male) with DCD that met the DSM‐5 (APA, 2013) diagnostic criteria: motor skills below the level expected for age and IQ, motor difficulties present early in developmental period and not explained by additional diagnoses. Of note, only two children scored at the 16th percentile on the MABC‐2, with the majority scoring ≤5th percentile (*n* = 17, 74%), and the remaining 5th–9th (*n* = 4, 17%).

Thirty CA matched children, aged 7‐10 years old, were recruited from primary schools in South London as a typically developing comparison group to the DCD group. Five children were excluded from further study due to scoring below the motor cut‐off. The final CA sample consisted of 25 children (19 male). The CA and DCD groups were comparable in age (*p* = .19; see Table 1).

Children in the MM group were recruited from the same schools as the CA group and screened based on the time it took them to complete the peg placing task that forms part of the MABC‐2. Children had to place 12 pegs into a board as quickly and accurately as possible, using their preferred (writing) hand and non‐preferred hand. Time taken (seconds) was recorded for two trials with each hand. The mean of the four trials was calculated and then matched to the overall group scores of children with DCD. While we recognize that matching on one single item from the MABC‐2 has its limitations, this method of ability matching has been used in another study (i.e. Sinani, Sugden & Hill, 2011). Children had to match the DCD group on peg placing performance and also demonstrate age‐appropriate motor skills to confirm that they were typically developing (i.e. a score ≥25th percentile on the relevant MABC‐2 age band). Twenty‐nine children (19 male), aged 4–7 years, met the full criteria for the MM group. The MM group was significantly younger than the DCD and CA groups (*p*s < .001), and no differences exist between the MM and DCD groups on mean peg placing scores (*p* = .68; see Table 1).

### Measures

#### Oculomotor battery

Eye movements were recorded at a sampling rate of 1000 Hz using the Eyelink 1000 eye tracker (SR‐research). The camera was positioned in the desktop mount position and a chin rest was used to keep the head stable. A 5‐point calibration was performed at the beginning of the testing session. This was repeated as required during the research session. The oculomotor battery consisted of four tasks presented in the order listed below (a–d). For all tasks, the stimuli/target consisted of a red circle presented against a black background on the computer screen with 1024 × 786 screen resolution. Children were seated directly in front of the computer monitor at a viewing distance of approximately 80 cm. The red circle measured 0.65° × 0.65° visual angle. Written instructions were displayed on the computer screen for each task and were also given verbally to all children. Breaks were factored in between tasks, if necessary.

- In the visual *fixation* task, children were instructed to maintain their gaze on the target that was shown in the centre of the screen, until it disappeared. The task began after a drift correct procedure and lasted 30 seconds.
- Children completed two *smooth pursuit* tasks, at differing speeds. Children were required to follow the target (i.e. keep their eyes on the target) which had a horizontal sinusoidal motion, moving at 0.2 Hz (slow trial) and then at 0.5 Hz (fast trial). Each trial lasted 20 s and was preceded by a drift correct procedure. The target travelled 8.5°/s in the slow trial and 21.5°/s in the fast trial.

Both the (c) prosaccade and (d) antisaccade tasks used the ‘step’ procedure, meaning that the cue disappeared at the same time as the peripheral target appeared. Each trial was preceded by a drift correct procedure which then moved on to display the central fixation target. The central target was displayed for 1000 ms before moving on the horizontal meridian 6.25° to the left or right. The direction of the step was randomized in both tasks and the target was displayed in the new location for 1000 ms. For the (c) *prosaccade* task, children completed 24 trials. They were instructed to look at the central fixation point and then move their eyes as quickly as possible to the target when it moved from the central point of the screen. For the (d) *antisaccade* task, the procedure remained the same (also 24 trials), but the instructions differed. Children were instructed to ignore the target when it moved from the centre of the screen and to look as quickly as possible in the opposite direction. The instructions were explained and then verified with the child. For example, the experimenter would point to a location on the screen (left or right) and say, ‘If the red circle moves this way, which direction should your eyes go?’ This was discussed until it was clear the child understood the requirements.

### Eye tracking data analysis

The experiment was implemented using Experiment Builder and analysed using Data Viewer (both SR Research software).

#### Fixation

The fixation task required sustained active engagement on the target. To determine whether this was achieved, three measures were taken to represent fixation ‘stability’: Time on Target (within 1° visual angle, represented as a %); Number of saccades during the 30 s task; Average fixation duration.

#### Smooth pursuit

Eye movements from the smooth pursuit trials were quantified using customized software written in LabView.^1^ For each potential pursuit segment, pursuit gain was calculated as the average of eye velocity divided by target velocity for all samples. Root‐mean‐square‐error (RMSE) was calculated as the square root of the average of eye position (in degrees of visual angle) subtracted from target position (in degrees of visual angle) squared. The duration of each segment was determined using the online parsing decisions made by the eye tracking software which classifies samples as being in a saccade if the sample velocity exceeds 30°/s, or acceleration exceeds 8000°/s. All samples not classified as being part of a saccade (or a blink – which includes periods of ‘tracking loss’) were considered as being in a potential pursuit segment, and velocity gain, duration and RMSE were calculated. Any pursuit segments with velocity gain values below 0.5 or above 1.5 were removed prior to analysis, as were pursuit segments less than 100 ms in duration, and with RMSE values of above 2. Low gain values and high RMSE values occur when participants make anticipatory saccades – these are typically followed by a stationary fixation (low velocity gain) at some distance ahead of the target (high RMSE). For each target speed, the average Gain and RMSE measures were weighted by the duration of pursuit segments. The analysis process provides four key metrics of smooth pursuit performance: Number of qualifying pursuit segments, Sum of durations of qualifying pursuit segments (i.e. time spent in pursuit), Weighted average velocity gain, Weighted average RMSE. Tracking loss (referring to when the eye was unable to determine the location of gaze, typically due to blinks or the participants turning away from the screen) was identified in both trials. Further analyses were conducted on the smooth pursuit data to determine the frequency of anticipatory and corrective saccades. Saccades that took the eye > 4° ahead of the target were identified and classed as ‘anticipatory’ (and those that were < 4° from the target were classed as ‘corrective’ (i.e. reducing position error).

#### Prosaccade and antisaccade

Trials were identified as valid if (1) the participant was fixating on the central fixation point at target onset and (2) the start time of the saccade was > 80 ms (i.e. not anticipatory). Only valid trials were considered in the analysis, which generated two main performance measures for both tasks: saccade latency (ms) and percentage of direction errors. Accuracy was also measured in the prosaccade task by considering amplitude (i.e. how close the eye lands to the target). Saccade amplitude was reported in degrees of visual angle by the eye tracking software. This calculation is based on the screen pixel co‐ordinates of the gaze data, using parameters for screen height, width, distance and pixel resolution.

### Statistical analysis

Parametric (ANOVA, ANCOVAs, MANCOVAs) and non‐parametric equivalents (Kruskal‐Wallis, Mann Whitney) were conducted to explore group differences. As children with DCD differed from the CA and MM groups on teacher ratings of hyperactivity, the SDQ hyperactivity measure is included as a covariate in subsequent between‐group analyses. To be able to conduct ANCOVAs on the oculomotor measures, any data that were highly skewed (not normally distributed) were log‐transformed. Finally, to determine which measures best predict group membership, a discriminant function analysis was conducted. Of note, there are four missing smooth pursuit data points for the DCD group (children of all ages and thus not influencing the mean age of the group or skewing ages). These recordings were unsuccessful, and missing information is clearly marked in the relevant tables.

### Procedure

This study was part of a larger project which was approved by Goldsmiths, University of London, ethics committee. Informed written consent was obtained from each parent and children gave verbal assent. For the DCD group, all assessments (motor and oculomotor) were completed either on the same day (with a sufficient break in between) or across two separate days. The typically developing groups completed the tasks at their school across two sessions. Children were seen individually in a quiet room (dimly lit for the eye tracking tasks) either at their school or the research lab.

## Results

The results section first focuses on identifying potential group differences on the oculomotor measures, and concludes by investigating which measures best differentiate between groups.

### Fixation performance

One‐way ANCOVAs (with the SDQ hyperactivity score as a covariate) were carried out on the fixation task measures (see Table 2). For all analyses, the covariate was not significant (all *p*s > .54). A significant effect of group was, however, evident for all fixation measures: the number of saccades made during the task, *F*(2, 72) = 5.25, *p* = .01, *n* ^2^_*p*_ = .12, average fixation duration, *F*(2, 72) = 3.86 *p* = .03, *n* ^2^_*p*_ = .09, and percentage of time spent fixating the target, *F*(2, 72) = 3.83, *p* = .02, *n* ^2^_*p*_ = .10. Bonferroni‐corrected comparisons revealed that children with DCD made significantly more saccades, had a lower average fixation duration, and spent less time on target that their CA peers (*p*s < .02); however, the DCD vs. MM comparison (*p*s >.34) and the CA vs. MM comparison (*p*s > .14) revealed similar performance on each measure. The mean scores highlight that the MM group scored between the DCD and CA groups, although the differences were not significant.

**Table 2:** Group comparisons for fixation and smooth pursuit performance

	CA (*n* = 25)	DCD (*n* = 23)	MM (*n* = 29)	Post hoc
*Fixation*				
No. of saccades				
Mean (*SD*)	22.52 (12.02)	37.04 (16.17)	31.10 (17.84)	DCD > CA, DCD=MM, CA=MM
Range	2–50	9–69	5–74	
Av. fixation duration (s)				
Mean (*SD*)	1.77 (1.93)	.92 (.71)	1.34 (1.16)	DCD < CA, DCD=MM, CA=MM
Range	.55–10	.28–3	.30–5	
Time on target (%)				
Mean	86%	67%	76%	DCD < CA DCD=MM, CA=MM
Range	40–99%	11–89%	10–99%	
*Smooth pursuit* a				
*Slow trial*				
No. of segments				
Mean (*SD*)	20.17 (5.61)	18.21 (6.13)	23.72 (5.30)	CA = DCD = MM
Range	11–30	9–28	16–36	
Pursuit duration (s)				
Mean (*SD*)	15.29 (2.13)	9.55 (2.97)	12.22 (2.43)	CA > MM > DCD
Range	10.13–18	3.58–14.81	7.65–16.92	
Average gain†				
Mean (*SD*)	1.02 (.07)	1.01 (.08)	.96 (.08)	CA = DCD = MM
Range	.88–1.16	.87–1.16	.82–1.09	
Average RMSE†				
Mean (*SD*)	.71 (.17)	.79 (.22)	.82 (.24)	CA = DCD = MM
Range	.43–.99	.46–1.30	.41–1.32	
*Fast trial*				
No. of segments				
Mean (*SD*)	27.96 (6.75)	14.74 (8.17)	22.79 (9.92)	CA > DCD, DCD=MM, CA=MM
Range	14–42	4–35	4–42	
Pursuit duration (s)				
Mean (*SD*)	11.45 (3.13)	5.04 (2.49)	8.00 (3.73)	CA> MM > DCD
Range	4.44–16.13	1.40–9.36	1.09–15.04	
Average gain†				
Mean (*SD*)	.98 (.08)	.91 (.11)	.87 (.08)	CA = DCD, DCD=MM, CA>MM
Range	.79–1.09	.72–1.12	.67–1.03	
Average RMSE†				
Mean (*SD*)	1.20 (.12)	1.25 (.19)	1.19 (.16)	CA = DCD = MM
Range	.95–1.44	.96–1.54	.87–1.42	

*Note*:

^a^Four missing data points for the DCD group on the smooth pursuit trials. ^†^Averages are weighted so that larger values contribute more than smaller values. s = seconds. Larger gain values = better pursuit; lower RMSE = better pursuit.

### Smooth pursuit performance

The smooth pursuit measures presented in Table 2 (number of segments, duration, gain and RMSE) are all based on segments of data during which actual smooth pursuit eye movements were occurring (e.g. the eye's velocity closely matched the target velocity). They do not include segments of data in which the eye was making corrective saccades (made during pursuit tasks to correct for increasing spatial error) or intrusive saccades (which take the eye away from the target) and the stationary fixations or very low gain pursuit which can occur after anticipatory saccades. Two separate MANCOVAs were carried out for the slow and fast smooth pursuit measures. The SDQ hyperactivity score was not a significant covariate (*p* = .50). Using Pillai's trace, there was a significant effect of group on performance on the slow trials, *V* = 0.56, *F*(8, 130) = 6.31, *p* < .001. Follow‐up separate univariate ANOVAs for the four measures employed a stricter *p*‐value (Bonferroni‐correction, *p* = .05/4 [therefore, *p* = .01]). Using this correction, non‐significant effects of group membership were evident for the number of pursuit segments, *F*(2, 67) = 3.38, *p* = .04, *n* ^2^_*p*_ = .10, pursuit gain, *F*(2, 67) = 3.67, *p* = .03, *n* ^2^_*p*_ = .10, and RMSE, *F*(2, 67) = 1.56, *p* = .22, *n* ^2^_*p*_ = .04. However, there was a significant effect of group on the duration of pursuit on the slow trial, *F*(2, 67) = 17.61, *p* < .001, *n* ^2^_*p*_ = .35. Post‐hoc analyses, shown in Table 2, revealed that children with DCD spent significantly less time in pursuit than both their CA peers (*p* < .001) and the MM group (*p* = .04), and that the CA group spent more time in pursuit than the MM group (*p* < .001).

On the faster trials, again the effect of the SDQ hyperactivity covariate was non‐significant (*p* = .78). Using Pillai's trace, there was a significant effect of group on performance on the fast trials, *V* = 0.46, *F*(8, 130) = 4.70, *p* < .001. Again the stricter *p*‐value was used (*p* = .01), revealing a significant effect of group on the number of pursuit segments, *F*(2, 67) = 6.11, *p* = .004, *n* ^2^_*p*_ = .15, duration of time in pursuit, *F*(2, 67) = 12.92, *p* < .001, *n* ^2^_*p*_ = .28, and gain, *F*(2, 67) = 9.71, *p* < .001, *n* ^2^_*p*_ = .23; but not for RMSE, *F*(2, 67) = .18, *p* = .84, *n* ^2^_*p*_ = .01. Post‐hoc analyses confirmed that children with DCD had significantly fewer segments of pursuit and a lower average duration of time in pursuit than the CA group (*p* = .003). Although, as above, the DCD and CA groups have similar gain and RMSE scores (*p*s > .19), the DCD group scored comparably to the younger MM group on all measures (*p*s > .15), while the MM group had a lower gain value than their older typically developing CA counterparts (*p*s < .001).^2^

**Figure 1:**
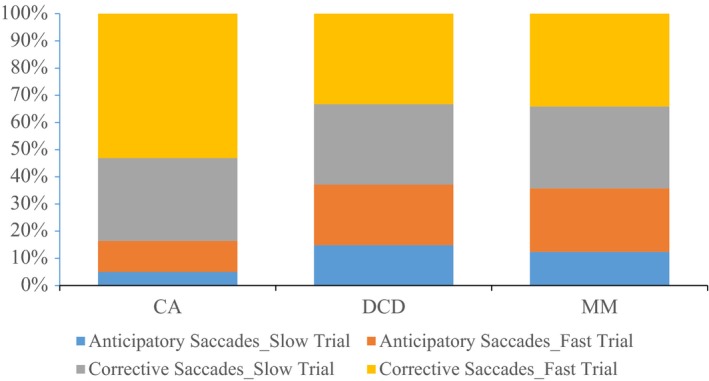
Proportion of large/anticipatory saccades (< 4° from the target) and small/corrective saccades (> 4° from the target) during the slow and fast smooth pursuit trials.

For descriptive purposes and to determine how the groups tracked the target when not in pursuit, the proportion of anticipatory (> 4° from the target) and corrective (< 4°) saccades in both pursuit trials are shown in Figure 1. It is apparent that children with DCD and the MM group made more anticipatory saccades in both trials, than the CA group.

### Prosaccade and antisaccade performance

Analyses were based only on valid trials, as discussed in the Methods data analysis section. The mean (*SD*) number of valid trials out of 24 on the prosaccade task per group were: DCD, 17.45 (3.82); CA, 21.00 (1.91); MM, 19.61 (4.39); and for the antisaccade task, also out of 24: DCD, 16.45 (4.75); CA, 19.08 (3.91); MM, 19.71 (4.91). The target moved 6.25° to the left or right of the centre, and analysis of prosaccades accuracy demonstrated that all groups performed similarly, *F*(2, 70) = .07, *p* = .93, with, on average, an amplitude very close to the target (DCD, *M* = 6.77°, *SD* = .73; CA, *M* = 6.81°, *SD* = .53; MM, *M* = 6.76°, *SD* = .88).

**Figure 2:**
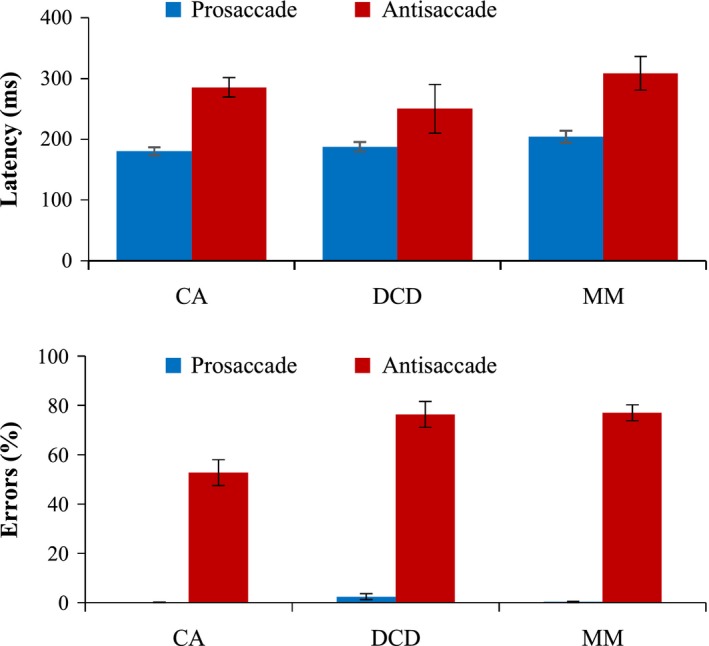
Mean saccade latency (in milliseconds) and percentage of errors made. Error bars represent standard error.

Figure 2 displays the average time it took the child to prepare (latency) a correct response (either towards the target (pro), or away from the target (anti)). A 3 × 2 (group × task) ANCOVA on latency scores revealed a significant effect of task, *F*(1, 69) = 8.04, *p* = .01, *n* ^2^_*p*_ = .10, showing that all children had faster latencies in the prosaccade than the antisaccade task (*p* < .001). A non‐significant effect of group, *F*(2, 69) = .95, *p* = .39, *n* ^2^_*p*_ = .03, and no interaction was found, *F*(2, 69) = .90, *p* = .41, *n* ^2^_*p*_ = .03. Hyperactivity was a non‐significant covariate (*p* = .75).

Percentage of direction errors was calculated relative to the number of valid trials per participant. Errors in the prosaccade task were minimal (one CA child made 1 error; two MM children made 1 error each; five children with DCD made errors, ranging from 1 to 4 errors only). Therefore, statistical analyses were conducted only on the antisaccade errors in Figure 2. Before this, first glance of the antisaccade errors revealed that five children with DCD and three in the MM group had a 100% error rate. This may have been because they found the task extremely difficult, or possibly because they misunderstood the instructions. To determine which explanation was more appropriate, ‘corrective’ shifts in gaze after the initial directional error were analysed. Shifts in gaze were only classed as ‘corrective’ if the eye moved at least 3.12° past the central point (9.37° in total) and, therefore, could not be considered to be simply returning to the central fixation/preparing for the next trial. Those children with a 100% error rate were only included in the antisaccade analyses if at least 60% of the directional errors were followed by a corrective shift – therefore occurring above chance and demonstrating that the participant clearly understood the instructions. After applying this cut‐off, one child with DCD and one in the MM group were excluded from the analysis (remaining: CA = 25, DCD = 24, MM = 28), suggesting that a substantial proportion of children with DCD had great difficulty inhibiting a saccade in this task. Even after log‐transformation the data were not normally distributed, therefore a Kruskal‐Wallis test^3^ was conducted. A significant group difference was found for antisaccade error rates *H*(2) = 14.86, *p* = .001. Mann‐Whitney tests highlighted that children with DCD made significantly more errors than the CA group, *U* = 130.00, Z = −3.10, *p* = .002, *r* = −.45, as did the MM group in comparison to the CA group, *U* = 146.00, Z = −3.51, *p <* .001, *r* = −.49; although the DCD and MM groups scored similarly, *U* = 279.00, Z = −.36, *p* = .72, *r* = −.05. Together the pro/antisaccade findings suggest that children with DCD did not have difficulty with response preparation but did show marked problems with response inhibition, in comparison to their age‐matched peers.

Table 3 provides a summary of the oculomotor findings.

**Table 3:** Summary of oculomotor findings

*Task*	*Group findings*
Fixation	Children with DCD have poorer fixation stability than their CA peers, evident by a larger number of saccades. The younger MM group perform similarly to both the DCD and CA groups.
Smooth pursuit	All groups are comparable in pursuit gain and RMSE (except for gain in the slow trial where the MM group score lower than the DCD and CA groups). Children with DCD spend less time (duration) in pursuit than the typically developing groups and make more anticipatory saccades during the task.
Prosaccade	All groups are comparable in response preparation (latency) and accuracy (amplitude), and make very few errors.
Antisaccade	All groups are comparable in response preparation (latency), although slower in this trial than on the prosaccades task. Children with DCD and the younger MM make more errors towards the target than the CA group.

### Oculomotor predictors of group membership

**Figure 3:**
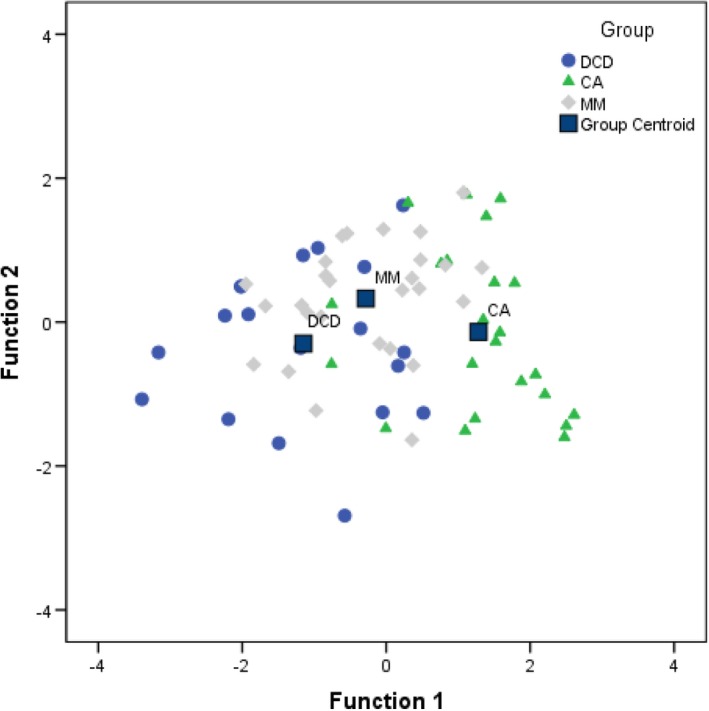
Canonical discriminant function graph.

Time spent on target and number of saccades made during the fixation trial, duration of smooth pursuit (slow and fast) and the measure of antisaccade errors were all included initially as predictor variables in a discriminant function analysis, as marked group differences were evident for each of these measures. The stepwise method was used to extract only the best predictors of group membership. Two discriminant functions were revealed. The first explained 92.8% of the variance, canonical *R* ^*2*^ = .49, whereas the second function explained only 9.2%, canonical *R* ^*2*^ = .07. These discriminant functions significantly differentiated the DCD, CA and MM groups, *Λ* = .47, *x* ^*2*^(4) = 49.75, *p* < .001. Correlations revealed that duration of smooth pursuit (slow trial) loaded highly onto both functions but more so for the first (*r* = .90 for the first function and *r* = .43 for the second); antisaccade error rate loaded more highly on the second function (*r* = .89) than the first function (*r* = −.45). The discriminant function plot (see Figure 3) showed that the first function discriminated the DCD and CA groups, and the second function discriminated the MM from the older DCD and CA groups.

## Discussion

This study aimed to determine whether oculomotor performance can distinguish between those with and without a general motor impairment. Both low level and higher order processes involved in oculomotor control were assessed. Several key findings emerged. Firstly, echoing the findings of Robert *et al*. (2014), we found that horizontal pursuit gain and RMSE were comparable across children with DCD and their age‐matched peers. Other measures of oculomotor integrity such as prosaccade latency and accuracy were also similar for these two groups. Together, these findings suggest that the fundamental neural mechanisms underlying pursuit and saccades are intact in DCD. In other words, when actively pursuing a target, children with DCD were able to do so as accurately as the controls, both in terms of matching their eye velocity to target velocity (gain) and in matching their gaze location with the target location (RMSE). Similarly, in the prosaccade task children with DCD could prepare a saccade as quickly and direct the end position of the saccade as accurately as the controls. It was noteworthy that across all measures small standard deviations were evident in the DCD group, emphasizing homogeneous group performance.

Detailed examination of the pursuit profile, however, identified considerable atypicality in the DCD group. Children with DCD spent far less time actually in pursuit in both the slow and fast trials, making more anticipatory saccades (overshooting the target) than the age controls. A similar finding of a high number of saccades made away from the target was also evident on the fixation task. Accurate performance on the fixation and smooth pursuit tasks requires the suppression of neurons responsible for saccadic movements that would move the eye away from, or ahead of, the target (Ross, Olincy, Harris, Radant, Adler *et al*., 1998). The large number of anticipatory saccades found in pursuit, and intrusive saccades in the fixation task, suggests that children with DCD may have difficulty with saccadic inhibition: a cognitive control problem (Ross *et al*., 1998). This idea is further confirmed by their poor performance on the antisaccade task which highlighted significant problems suppressing prosaccades (response inhibition), in comparison to the CA group. While tracking loss was minimal and the measure of hyperactivity did not emerge as a significant co‐variate in any analyses, a higher number of saccades would not completely account for children with DCD spending nearly half as much time in pursuit as their peers. Difficulties with sustained attention (i.e. the overt orientation of visual attention toward the target) may partly explain the fixation and smooth pursuit results too. Thus, the findings are suggestive of difficulty exerting top‐down cognitive control.

Inclusion of the MM group sheds light on whether differences found between the DCD and CA groups are related to immaturity or atypical development. Difficulties with saccadic inhibition were apparent in the typically developing MM group who were comparable to the DCD group in anticipatory saccades during pursuit and antisaccade performance. On the other hand, in comparison to the MM group, children with DCD had larger deficits in pursuit duration and, to a lesser extent, in fixation stability. Therefore, while immature in some respect, children with DCD have more pronounced difficulties with top‐down control that requires maintaining attention, even compared to younger children. This finding is particularly striking given that higher order control (i.e. executive skill) is typically better in older children (Johnson & de Haan, 2015). It was also noteworthy that the CA and MM groups performed similarly on all measures except for duration of pursuit and antisaccade error rates, for which the CA outperformed their younger counterparts, confirming that these measures are linked to maturation in typically developing populations (Ingser‐Moati *et al*., 2009). In terms of potential group markers, indicated by function 1 of the discriminant function analysis, the duration of pursuit (slow trial) successfully differentiated between children of the same age with and without a motor impairment (DCD vs. CA), while antisaccade error rate loaded highly on function 2 and differentiated children of different ages (MM vs. CA and DCD).

The smooth pursuit findings do not appear to support the internal modelling deficit hypothesis (i.e. difficulties with predictive control) of DCD (Adams *et al*., 2014), since difficulty in this respect should result in poor gain/RMSE during pursuit with a subsequent increase in corrective saccades. In fact, we find generally good pursuit and an increase in anticipatory saccades during the smooth pursuit tasks, which suggests a general inability to maintain attentional focus on the target. The specific oculomotor impairments reported here contribute more so to better understanding the neurological phenotype of DCD. The pursuit system is under the control of various cerebral structures: the cerebellum, pontine nuclei, central thalamus, medial superior temporal cortex, and supplementary and frontal eye fields (SEF and FEF), and poor pursuit gain is thought to result from cerebellar dysfunction (Robert *et al*., 2014; Thier & Ilg, 2005). The cerebellum is frequently referenced in DCD (Wilson *et al*., 2013; Zwicker, Missiuna & Boyd, 2009). However, studies reporting soft neurological signs (i.e. poor coordination, delayed motor milestones) and associating these with specific neurological structures such as the cerebellum should be treated with caution, as these characteristics can also be found in children with no motor difficulties (Cantin, Polatajko, Thach & Jaglal, 2007). The present study empirically tested smooth pursuit whereby the cerebellum plays a crucial role and the findings, as do Robert *et al*. (2015), argue against the idea that DCD reflects a cerebellar disturbance. Rather, deficits in the fronto‐parietal circuit have been associated with poor planning of saccadic movements and saccadic disinhibition (Camchong, Dykman, Austin, Clementz & McDowell, 2008; Miller, Sun, Curtis & D'Esposito, 2005; Ross *et al*., 1998) and may be linked to the profile of performance in the present study.

The findings also contribute to the existing literature on executive control (Bernardi *et al*., 2015; Leonard & Hill, 2015) by confirming a specific difficulty in inhibition even when motor and verbal demands are eliminated. Neural correlates of antisaccade tasks are widespread, with increased activity evident in the SEF, FEF, dorsolateral prefrontal cortex (DLPFC), anterior cingulate cortex, basal ganglia and cerebellum (De Souza, Menon & Everling, 2003). Therefore, pinpointing specific areas of ‘dysfunction’ can be difficult; and the parietal lobe, DLPFC, basal ganglia and cerebellum are all believed to be implicated in DCD (Peters, Maathuis & Hadders‐Algra, 2013; Zwicker *et al*., 2009). Future research combining fMRI techniques and investigation of saccadic eye movements in DCD would prove fruitful. In a practical sense though, inefficient development of the neural circuits that exert top‐down control over motor actions, for example inhibition and directing visual attention, could interfere with the acquisition and refinement of general motor skill (Johnson & de Haan, 2015). For example, in ball games, saccades are rapid in response to tracking a ball, and smooth pursuit will take over when tracking a slow moving object. If children have difficulty staying focused on task (i.e. suppressing intrusive saccades that may be a response to distractions in the environment), it is understandable that they will miss crucial visual information and, as a result, have difficulty coordinating actions successfully. Future research would benefit from using eye tracking technology in real‐world motor learning tasks to further understand this relationship.

While this study addressed a number of limitations of the two previous oculomotor studies, it had limitations of its own. Mixed findings have been reported with regard to general visual deficits in DCD. Some find no differences between children with DCD and their peers (Mon‐Williams, Pascal & Wann, 1994), while others using population‐based samples find that children with more ‘severe’ motor difficulties are at higher chance of having reduced stereoacuity and hypermetropia (Creavin, Lingam, Northstone & Williams, 2014). Assessment of general visual deficits were beyond the scope of the present study and while we screened for known visual impairments via the parent questionnaire, we acknowledge that future research may benefit from including visual acuity, or similar measures, as a screening tool alongside oculomotor performance. Moreover, inclusion of frequently co‐occurring disorders such as ADHD would be advantageous to enhance the representative nature of a DCD sample. Given reports of deficits in saccade response preparation *and* inhibition in children with ADHD (Mahone *et al*., 2009), we might expect that children with a dual diagnosis (DCD+ADHD) would present with more pronounced difficulties than reported here in the DCD‐only sample. Notably we identify difficulties with sustained overt visual attention, which could be indicative of broader attentional problems even if these children do not meet the criteria for an ADHD diagnosis. Future research is warranted to consider the overlap of attention and motor problems and the extent to which the dimensional aspect of attention difficulties may contribute to the findings.

In summary, our findings suggest that children with DCD have marked problems with sustained engagement on fixation and smooth pursuit tasks. Pronounced difficulties with suppressing reflexive saccades were apparent in both a DCD and younger typically developing group. However, the underlying mechanisms of pursuit and saccades were found to be comparable to typically developing controls, meaning that fundamental oculomotor processes are intact in DCD. Duration of pursuit and antisaccade performance were identified as measures that can differentiate children with and without a motor impairment, as well as differing age groups. As eye tracking methodologies become less intrusive, they are becoming increasingly popular in research on neurodevelopmental disorders. Careful examination of eye movements furthers our understanding of the neurological phenotype in DCD and may provide objective markers to aid earlier identification and avenues for intervention approaches.

## Notes

1 Software is available on request.

2 Of note, on the slower trial, *F*(2, 68) = 4.20, *p* = .02, *n* ^2^_*p*_ = .11, tracking loss was comparable for the DCD vs. CA and DCD vs. MM groups (*p*s > .48), but the CA group had more loss than the MM group (*p* = .02): ranging from 1.37 to 1.94 seconds; while tracking loss was equivalent across all groups, *F*(2, 68) = .77, *p* = .47, *n* ^2^_*p*_ = .02, on the fast speed trial: ranging from .79 to 1.03 seconds.

3 No ANCOVA equivalent for non‐parametric tests. However, when an ANCOVA was run (although violating assumptions of normality) hyperactivity was not a significant covariate (*p* = .11) and so exclusion of the covariate from this particular analysis was not considered problematic.
